# Supplementary figures and images for: Coffee intake is associated with telomere length in severe mental disorders
Source: BMJ Ment Health. 2025 Nov 25;28(1):e301700. doi: 10.1136/bmjment-2025-301700 (PMC12658549; doi:10.1136/bmjment-2025-301700)

**Figure S1.** Flowchart of participants exclusion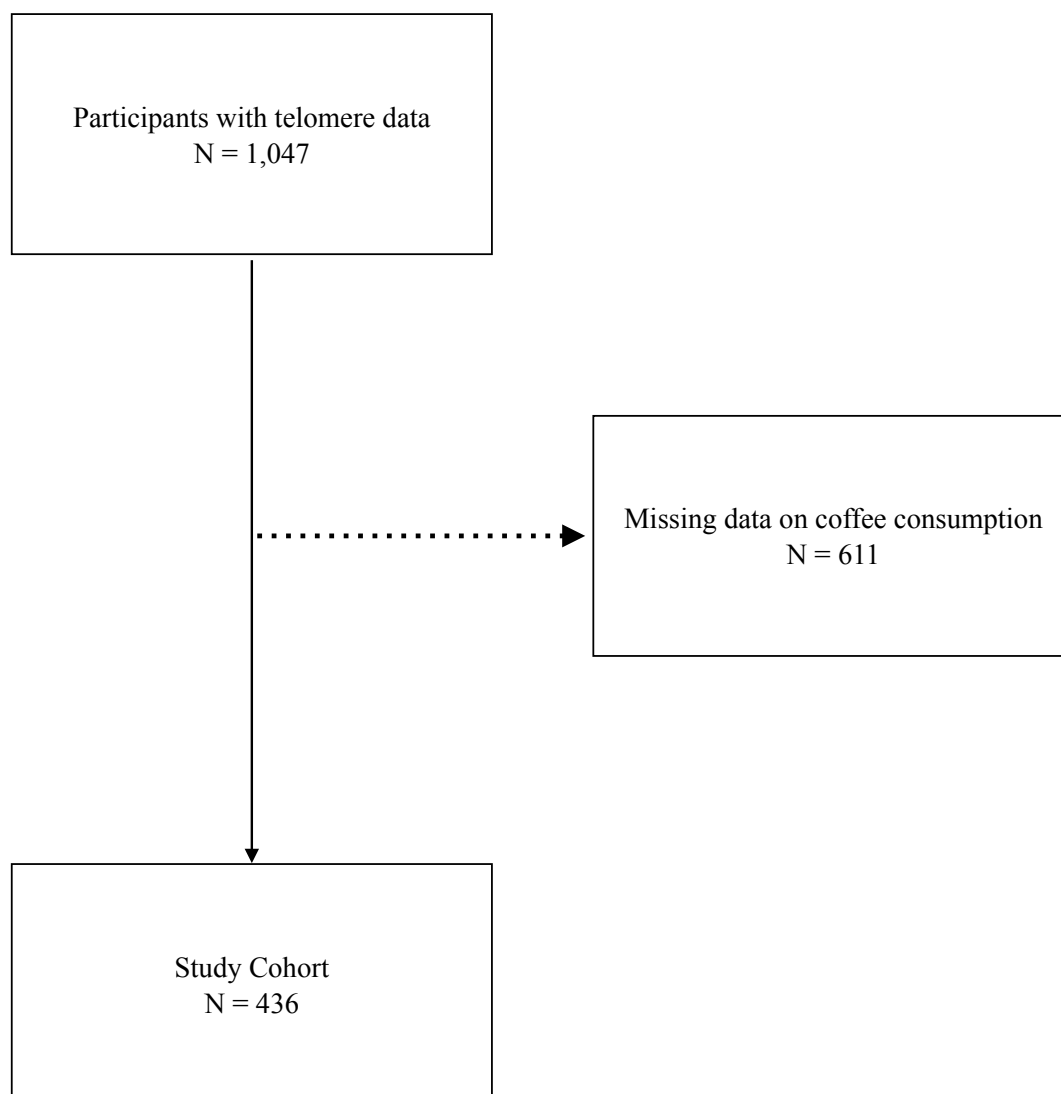

Supplement: Supplementary data [file bmjment-28-1-s001.pdf]
